# Association between the Polymorphism rs3217927 of CCND2 and the Risk of Childhood Acute Lymphoblastic Leukemia in a Chinese Population

**DOI:** 10.1371/journal.pone.0095059

**Published:** 2014-04-17

**Authors:** Heng Zhang, Yan Zhou, Yaoyao Rui, Yaping Wang, Jie Li, Liuchen Rong, Meilin Wang, Na Tong, Zhengdong Zhang, Jing Chen, Yongjun Fang

**Affiliations:** 1 Department of Hematology and Oncology, Nanjing Children's Hospital Affiliated to Nanjing Medical University, Nanjing, China; 2 Department of Hematology and Oncology, Soochow Children's Hospital Affiliated to Soochow University, Suzhou, China; 3 Department of Molecular and Genetic Toxicology, Cancer Center of Nanjing Medical University, Nanjing, China; 4 Department of Hematology and Oncology, Shanghai Children's Medical Center Affiliated to Shanghai, Jiao Tong University, Shanghai, China; Westmead Millennium Institute, University of Sydney, Australia

## Abstract

CyclinD proteins, the ultimate recipients of mitogenic and oncogenic signals, play a crucial role in cell-cycle regulation. CyclinD2, one of the cyclinD family, is overexpressed in T-acute lymphoblastic leukemia (ALL) and B-cell chronic lymphocytic leukemia and involved in the pathogenesis of leukemias. Recent reports indicated that *CCND2* polymorphisms are associated with human cancer risk, thusly we hypothesized that *CCND2* gene polymorphisms may contribute to childhood ALL susceptibility. We selected the polymorphism rs3217927 located in the 3′UTR region of *CCND2* to assess its associations with childhood ALL risk in a case-control study. A significant difference was found in the genotype distributions of rs3217927 polymorphism between cases and controls (P = 0.019) and homozygous GG genotype may be an increased risk factor for childhood ALL (adjusted OR  =  1.84, 95% CI  =  1.14 —2.99). Furthermore, this increased risk was more pronounced with GG genotype among high-risk ALL (adjusted OR  = 1.95, 95% CI  =  1.04–3.67), low-risk ALL (adjusted OR  =  2.09, 95% CI  =  1.13–3.87), B-phenotype ALL patients (adjusted OR  =  1.78, 95% CI  =  1.08–2.95) and T-phenotype ALL patients (adjusted OR  =  2.87, 95% CI  =  1.16–7.13). Our results provide evidence that *CCND2* polymorphism rs3217927 may be involved in the etiology of childhood ALL, and the GG genotype of rs3217927 may modulate the genetic susceptibility to childhood ALL in the Chinese population. Further functional studies and investigations in larger populations should be conducted to validate our findings.

## Introduction

Pediatric cancer is now the second most common cause of death by disease in children in developed countries [1]. Acute lymphoblastic leukemia (ALL), the most common childhood malignancy, accounts for 26.8% of all pediatric cancers among children less than 15 years of age, with approximately 38 new cases per million children diagnosed with ALL each year in the developed world [2]. The peak incidence of childhood ALL occurs at age 2 to 5 years, suggesting that ALL may initiate in utero or during the infant period [2], [3]. In recent decades, cure rate for childhood ALL have increased from 10% to nearly 85% due to optimized diagnosis, risk stratification, and chemotherapy protocols [4]. Nevertheless, childhood ALL remains a leading cause of cancer death in children. Understanding the potential etiologies of childhood ALL to reduce the incidence of pediatric ALL remains crucial.

It is widely accepted that the interaction between genes and the environment contributes to the pathogenesis of ALL in children. Potential molecular mechanisms implicated include sequential alterations in tumor-suppress genes, proto-oncogenes, and microRNA genes of hematopoietic stem cells or their committed progenitors [5]. Multiple recent studies indicate that genetic polymorphisms play an influential role in childhood ALL susceptibility, treatment response, and prognosis [6].

D-type cyclins, the ultimate recipients of mitogenic and oncogenic signals, have been recently recognized as potentially suitable molecular targets for chemotherapy due to their specific over-expression in malignancies [7]–[9]. The D-type cyclins (cyclinsD1, cyclinsD2, cyclinsD3) are separately encoded by *CCND1*, *CCND2* and *CCND3*. *CCND2* is one of the direct transcriptional targets of Hedgehog signaling and PI3K-AKT signaling pathways [10], [11]. Abnormal activation of these two pathways leads to disorders of cell proliferation, metabolism, growth and survival, and has been proven implicated in hematologic malignancies [8], [9], [12]. Progression of the cell cycle is modulated via the sequential and well-ordered activation of several components. CyclinD2, one of the key components, plays an important role in regulating the G1/S transitions in cell cycle [13]. The abnormal expression of cyclinD2 may influence the repair of DNA damage and initiate of tumorigenesis, including leukemogenesis [14]. Multiple studies of inherited variation in cell cycle genes suggest that genotypes in this pathway may be associated with increased risk of breast cancer, prostate cancer, lung cancer, bladder cancer and oral cancer [15]–[21]. Recently, a genome-wide association study (GWAS) showed the *CCND2* inherited genetic variations are associated with the risk of colorectal cancer in Europeans and Asians [22], [23]. However, few studies have detected the effects of *CCND2* gene polymorphisms on the risk of childhood ALL.

To detect the potential impact of *CCND2* polymorphisms on the risk of developing childhood ALL in a Chinese population, we selected a tagging single nucleotide polymorphism (tSNP) rs3217927 from the data for Chinese in the HapMap database (http://hapmap.ncbi.nlm.nih.gov/), which locates in the 3′ untranslated region (UTR) of *CCND2*. We then genotyped this SNP in 753 patients with childhood ALL and 1088 normal controls in this case control study.

## Materials and Methods

### Study subjects

The research protocol was approved by the Medical Ethics Committee of Nanjing Children's Hospital affiliated to Nanjing Medical University. Written informed consent was obtained from the parents or legal guardians of all study subjects. From January 2007 to January 2013, 857 subjects with newly diagnosed childhood ALL and 1151 cancer-free controls were recruited from the Nanjing Children's Hospital Affiliated Nanjing Medical University, Shanghai Children's Medical Center Affiliated to Shanghai Jiao Tong University and Soochow University Affiliated Children's Hospital. Parents or legal guardians of the all subjects were interviewed in person to collect demographic data and exposure information, including age, gender, parental alcohol use and tobacco-smoking and painting status of the home. Then the numbers of subjects were reduced into 753 cases and 1088 controls because of the missing of demographic data. The 753 cases included 291 females and 462 males, with a median age of 5 years. Subjects aged from 1 to 18 years and were genetically unrelated to ethnic Han Chinese. All subjects underwent bone marrow aspiration and the diagnosis of ALL was confirmed by morphology, immunohistochemistry, cytogenetics, and molecular biology. Risk stratification of the cases was determined uniformly according to the Suggestion of Diagnosis and Treatment of ALL in Childhood, published by the Society of Pediatrics, Chinese Medical Association in 2006. The control subjects had no history of malignant neoplasm or thrombotic disease and were age and gender matched to the cases.

### Genotyping

Genomic DNA was extracted by standard protocols (Qiagen) from isolated peripheral blood lymphocytes. The *CCND2* rs3217927 was genotyped with 384-well ABI 7900HT Real-Time PCR System (Applied Biosystems, Foster City, CA) using TaqMan SNP Genotyping assay. The reaction contained 5 ng genomic DNA, TaqMan Master Mix, forward and reverse primers and probes for the wild type and the mutant allele in a total volume of 5 ul. The primer sequences were 5′-CTGCGCAGGCAAGCACTAT-3′ and 5′-CCTGCCAATTCAGTGTGATTGA-3′, and the probes were 5′-FAM-CTCTGCTGAGCGGTA-MGB-3′ and 5′-HEX-CCTCTGCTAAGCGG-MGB-3′, which were devised and manufactured by Nanjing Steed Biotechnology (Nanjing, China). The location of the CCND2 primer sequence is shown in Figure S1. The PCR thermal cycling amplification was performed under the following conditions: 95°C for 10 min followed by 45 cycles of 95°C for 15 s and 60°C for 1 min. The genotype analysis was done blinded and 10% of control samples were randomly repeated for the typing reliability, proven complete concordant.

### Expression levels of *CCND2* mRNA

We extracted total RNA from bone marrow in 57 ALL patients from total 753 cases randomly. The total RNA was reverse transcribed into complementary DNA using ReverTra Ace qPCR RT kit (Toyobo, Tsuruga, Japan), and the complementary DNA was used for subsequent real-time PCR analysis (ABI 7300). Glyceraldehyde 3-phosphate dehydrogenase (GAPDH) was used as an internal quantitative control for each sample and each assay was done in triplicate. The primers used for amplification were 5′-TCATTGCTCTGTGTGCCACC-3′and 5′-CAGCTCAGTCAGGGCATCAC-3′ for *CCND2*, and 5′-GCACCGTCAAGGCTGAGAAC-3′ and 5′-GGATCTCGCTCCTGGAAGATG-3′ for GAPDH. The PCR thermal cycling protocol consisted of 50°C for 2 min, then 95°C for 10 min followed by 45 cycles of 95°C for 15 s and 60°C for 1 min.

### Statistical analysis

Hardy–Weinberg equilibrium of the genotype distribution for each SNP among the control group was examined by a goodness-of-fit χ2test. Chi-square (χ2) test was used to evaluate the distribution differences of selected demographic characteristics as well as each allele and genotypes of rs3217927 between the cases and controls. Unconditional univariate and multivariate logistic regression analyses were performed to obtain crude and adjusted odds ratios (ORs) for estimating risk of childhood ALL and their 95% confidence intervals (CIs) with adjustment for diagnosis age, gender, parental alcohol use, tobacco-smoking and housing painting status. Independent-sample *t*-test was used for analyzing the results of *CCND2* mRNA expression. Two-sided P values were selected and P<0.05 was considered statistically significant. All the statistical analyses were performed using Statistics Analysis System software (version 9.1; SAS Institute, Cary, NC).

## Results

### Characteristics of the study subjects

The frequency distributions of all subjects including selected demographic variables are summarized in Table 1. The median age of the recruited subjects was used as the age stratification standard. There was no significant difference in the distribution of age (P = 0.20) and sex (P = 0.89) and smoke (P = 0.08) between the cases and controls. However, compared with the control subjects, the cases had more parental drinkers and home painting exposure during the pregnancy of mother or after the birth (34.5% versus 21.0%, P<0.001; 35.9% versus 26.2%, P<0.001, respectively). Of the 753 ALL patients, 95 (12.6%) were T-ALL, 655 (87.0%) were B-ALL; the others (0.4%) were T-B cell biphenotypic acute lymphoblastic leukemia. Furthermore, 297 (39.4%) patients were in the low-risk, 163 (21.7%) patients were in the medium-risk and the remaining 293 (38.9%) were in the high-risk. All the variables above were further adjusted in the multivariate logistic regression analysis.

**Table 1:** Frequency distribution of selected variables between cases of childhood ALL and cancer-free controls.

	Cases (n = 753)		Controls (n = 1088)		
Variables	n	%	n	%	*P* a
Age (years)					
≤5	404	53.65	551	50.64	0.204
>5	349	46.35	537	49.36	
Gender					
Male	462	61.35	671	61.67	0.890
Female	291	38.65	417	38.33	
Parental smoking status					
Never	314	41.70	498	45.77	0.084
Ever	439	58.30	590	54.23	
Parental drinking status					
Never	493	65.47	860	79.04	<.0001
Ever	260	34.53	228	20.96	
House-painting status					
Never	483	64.14	803	73.81	<.0001
Ever	270	35.86	285	26.19	
Immunophenotype					
B-ALL	655	86.98	-	-	-
T-ALL	95	12.62	-	-	-
Other^b^	3	0.40	-	-	-
Treatment branch					
Low risk	297	39.44	-	-	-
Medium risk	163	21.65	-	-	-
High risk	293	38.91	-	-	-

ALL, acute lymphoblastic leukemia; B-ALL, B-phenotype ALL; T-ALL, T-phenotype ALL; –, data not essential.

a Two-sided chi-square test for either genotype distribution or allele frequencies between cases and controls.

b Represents T-B cell biphenotypic acute lymphoblastic leukemia and other immunophenotypes.

### Association between *CCND2* polymorphism and ALL risk

The observed genotype distributions and allele frequencies for *CCND2* rs3217927 polymorphism in cases and controls and their associations with childhood ALL risk are presented in Table 2. The genotype frequencies of rs3217927 polymorphism among the controls were in agreement with Hardy–Weinberg equilibrium (p = 0.461). As shown in Table 2, the frequencies of AA, AG and GG genotypes were 61.5%, 32.9%, and 5.6%, among the cases, and 65.4%, 31.6%, and 3.0%, among the controls, respectively. There was a statistically significant difference in the genotype distributions of the *CCND2* rs3217927 polymorphism between the cases and controls (P = 0.011).

**Table 2:** Logistic regression analysis of association between the rs3217927 polymorphisms and the risk of childhood ALL.

Genotypes	Cases (n = 753)		Controls (n = 1088)		*Crude OR* (95% *CI*)	*Adjusted OR* (95% *CI*)^b^	*P* a
rs3217927	n	%	n	%			
**A>G**							
**AA**	463	61.5	712	65.4	1.00 (reference)	1.00 (reference)	0.019
**AG**	248	32.9	344	31.6	1.11 (0.91 1.36)	1.14 (0.93 1.39)	0.222
**GG**	42	5.6	32	3.0	2.02 (1.26 3.24)	1.84 (1.14 2.99)	0.013
**AG/GG**	290	38.5	405	34.6	1.19 (0.98 1.44)	1.20 (0.99 1.46)	0.070
**AA/AG**	711	94.4	1056	97.0	1.00 (reference)	1.00 (reference)	0.021
**GG**	42	5.6	32	3.0	1.95 (1.22 3.12)	1.76 (1.10 2.85)	
**G allele**		0.22		0.19			0.599

a Two-sided chi-square test for either genotype distribution or allele frequencies between cases and controls.

b Adjusted for age, gender, parental drinking status, parental smoking status, and house painting status.

Multivariate logistic regression analysis revealed that the homozygous GG genotype of rs3217927 was associated with an increased risk of childhood ALL compared with the homozygous AA genotype (adjusted OR  =  1.84, 95% CI  =  1.14 —2.99), while the heterozygous AG was not (adjusted OR  =  1.14, 95% CI  =  0.93–1.39). We also observed an elevated risk in the recessive model of *CCND2* rs3217927 polymorphisms (adjusted OR  =  1.76, 95% CI  =  1.10–2.85), but not in the dominant model (adjusted OR  =  1.20, 95% CI  =  0.99–1.46).

### The stratified analysis of the associations between rs3217927 polymorphism and clinical features of ALL

The further stratification analysis was developed to evaluate the risk of *CCND2* rs3217927 polymorphism and some clinical variables of ALL. As shown in Table 3, the increased risk was more pronounced with GG genotype among high-risk ALL (adjusted OR  =  1.95, 95% CI  =  1.04–3.67), low-risk ALL (adjusted OR  =  2.09, 95% CI  =  1.13–3.87), B-lineage ALL (B-ALL) patients (adjusted OR  =  1.78, 95% CI  =  1.08–2.95) and T-lineage ALL(T-ALL) patients (adjusted OR  =  2.87, 95% CI  =  1.16–7.13). In contrast, with the recessive model, the increased risk was found only in low-risk ALL (adjusted OR  =  2.05, 95% CI  =  1.12–3.76), B-phenotype ALL patients (adjusted OR  =  1.71, 95% CI  =  1.03–2.82) and T-phenotype ALL patients (adjusted OR  =  2.53, 95% CI  =  1.04–6.19). No significant associations were observed between the genotypes and risk of medium-risk ALL.

**Table 3:** Association of rs3217927 (CCND2) polymorphism with clinical risk and immunophenotype of childhood ALL.

Genotype	Controls		Clinical risk								*OR* (95% *CI*)^a^			Immunophenotype^b^					*OR* (95% *CI*)^a^	
	n = 1088		High n = 293			Medium n = 163			Low n = 297		High	Medium	Low	B-ALL (n = 655)			T-ALL (n = 95)		B-ALL	T-ALL
	n	%	n	%		n	%		n	%				n	%		n	%		
**AA**	712	65.4	175	59.7		99	60.7		189	63.6	1.00 (reference)	1.00 (reference)	1.00 (reference)	406	62.0		54	56.8	1.00 (reference)	1.00 (reference)
**AG**	344	31.6	102	34.8		56	34.4		90	30.3	1.21 (0.92 1.61)	1.23 (0.86 1.76)	1.07 (0.80 1.42)	214	32.7		34	35.8	1.14 (0.92 1.41)	1.33 (0.84 2.10)
**GG**	32	3.0	16	5.5		8	4.9		18	6.1	1.95 (1.04 3.67)	1.58 (0.70 3.58)	2.09 (1.13 3.87)	35	5.3		7	7.4	1.78 (1.08 2.95)	2.87 (1.16 7.13)
**AG/GG**	405	34.6	118	40.3		64	39.3		108	36.4	1.28 (0.96 1.67)	1.26 (0.90 1.78)	1.16 (0.88 1.53)	249	38.0		41	43.2	1.20 (0.97 1.47)	1.45 (0.94 2.24)
**AA/AG**	1056	97.0	277	94.5		155	95.1		279	93.9	1.00 (reference)	1.00 (reference)	1.00 (reference)	620	94.7		88	92.6	1.00 (reference)	1.00 (reference)
**GG**	32	3.0	16	5.5		8	4.9		18	6.1	1.78 (0.95 3.34)	1.49 (0.66 3.35)	2.05 (1.12 3.76)	35	5.3		7	7.4	1.71 (1.03 2.82)	2.53 (1.04 6.19)

a *OR*s and 95% *CI*s were calculated by logistic regression analysis.

b There were three ALL patients diagnosed with B+T-ALL and they were all AA genotype.

### Association between *CCND2* rs3217927 polymorphism and the expression levels of *CCND2* mRNA

**Figure 1:**
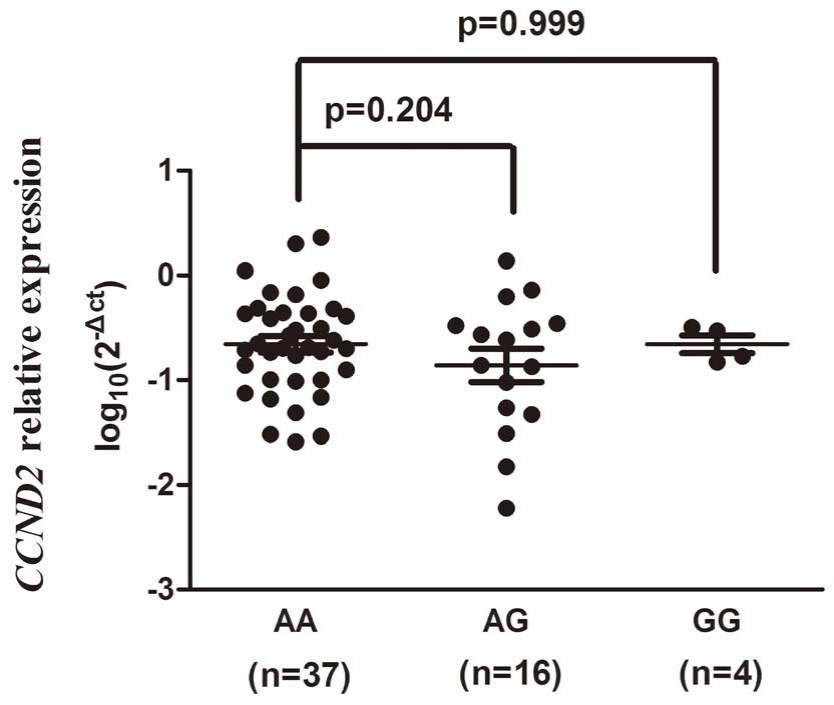
Association between the rs3217927 polymorphism and relative CCND2 mRNA expression. The frequency distributions of the AA, AG and GG genotypes were 37, 16 and 4, respectively. The fold change was normalized against GAPDH. P = 0.204 for AG compared with AA and P = 0.999 for AG compared with AA.

**Figure 2:**
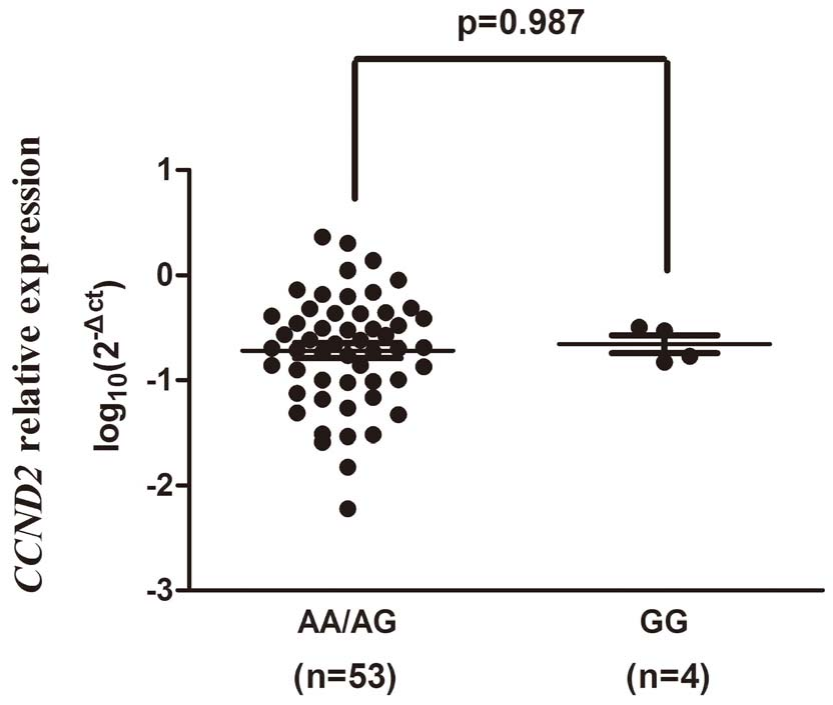
Association between the recessive model of rs3217927 polymorphism and relative CCND2 mRNA expression. The frequency distributions of the AA/AG and GG genotypes were 53 and 4, respectively. P = 0.987 for GG compared with AA/AG.

57 ALL patients with three genotypes of CCND2 (37 patients with AA genotype, 16 patients with AG genotype and 4 patients with GG genotype, respectively) were performed here. As shown in Figure 1 and Figure 2, no obvious difference was found here that A to G mutation changed the expression level of CCND2 mRNA. The expression levels of the AG carriers (P = 0.204) and the GG carriers (P = 0.999) has no significant difference with those of AA carriers. We than conduct a recessive model, no statistical significance has been found, either (P = 0.987).

## Discussion

This ongoing study explored the association of *CCND2* rs3217927 polymorphism in 753 newly diagnosed childhood ALL patients and 1088 cancer-free controls. We found that the G allele of *CCND2* polymorphism rs3217927 has a significant association with childhood ALL in a Chinese population and may be a risk factor for the development of the disease. In the stratified analysis, strong evidence were found to prove a pronounced association between homozygous GG and high-risk ALL, low-risk ALL, T-phenotype and B-phenotype ALL sub-groups, thus putting the homozygous GG a potential risk factor for ALL. As shown in Table 1, the epidemiologic data indicates that the cases had more parental alcohol consumption and house painting exposure during the pregnancy or after the birth than the controls, indicating a possible association between parental alcohol use and paint exposure with childhood ALL. Compared with adults, children may be at higher risk of environmental toxicants due to exposure differences, physiologic immaturity, higher cell growth and proliferation [24], [25], although there were no observably significant statistical interactions between environmental exposure factors and the polymorphism, which is shown in Table S1.

The human *CCND2* gene, located on chromosome 12p13.32 and spanning 31.6 kb, is a necessary member of the cyclinD gene family which promotes cell growth and proliferation [26]. Once induced by extracellular mitogenic environment, D cyclins will bind and activate the relative cyclin-dependent kinases CDK4 and CDK6 to form the Cyclin/CDK complexes. These complexes promote the cell cycle crossing the restriction-point and subsequently entering the phase of DNA synthesis [26]. In normal mammalian cells, the so-called check-point controls are the G1-to-S and G2-to-M transitions. The progress of cell cycle can stop and delay at these restriction-points to permit cell repairing of damaged DNA and prevent various types of mutations [27]. Over-expression of cyclinD shortens the duration of the G1-phase [14], thereby impeding the repair of damaged DNA, and may result in the development of cancer. The over-expression of cyclin D2 has been observed in many tumors including acute leukemia, and may contribute to unlimited cell division, prevention of programmed death and chemoresistance[28]. Compared with unaffected people, cyclin D2 exhibited over-expression in B-cell chronic lymphocytic leukemia cases and preferentially expressed in human T-lymphotropic virus type I (HTLV-I) infected T cell lines [28], [29]. Karrman K and Clappier E *et al*. discovered deregulation of cyclinsD2 by translocational activation in T-cell acute lymphoblastic leukemia [30], [31]. Xinliang Mao *et al* demonstrated cyclinsD2 was a therapeutic target of myeloma and leukemia in mouse models and believed decreased D-cyclins may be a biomarker of an anticancer effect [32]. Recent related work declared *CCND2* inherited genetic variations linked with the risk of colorectal cancer and ovarian cancer [22], [33]. However, Sheng H *et al*. reported no significant association had been found between *CCND2* rs3217927 and non-small cell lung cancer in a Chinese population [34]. No reports have found the relationship between genetic variations in *CCND2* and childhood ALL.

In this study, the relevance of tagSNP rs3217927 of *CCND2* and childhood ALL risk was investigated. We found that the homozygous GG genotype of the *CCND2* rs3217927 correlated with a significantly increased risk of childhood ALL. This increased risk was most pronounced in high-risk ALL, low-risk ALL, B-phenotype and T-phenotype ALL patients, but not medium risk group, which may due to the lower number of samples in medium risk group reducing statistical power. A combination between AA genotype and AG genotype is performed as reference to conduct a recessive model, and the homozygous GG genotype still showed a significantly increased risk of childhood ALL among low-risk, B-phenotype and T-phenotype ALL patients, which indicated the importance of the association between homozygous GG and the risk of childhood ALL. Furthermore, the high risk of more parental alcohol consumption and house painting exposure, as well as genetic susceptibilities in rs3217927GG genotype in the subgroup, were observed in our study. The result above indicated ALL formation might be subjected to a variety of environmental expose and genetic factors. However, according to recent GWAS, the association between *CCND2* polymorphisms and childhood ALL risk was not observed, as a possible result of ethnic differences [35]–[38].

It has been reported that the binding affinity between miRNA and its target mRNA may be changed by the SNPs located in miRNA target sites. The difference of those SNPs may lead to degradation of the mRNA and inhibition of the mRNA translation into proteins [39]. For further functional studies of this association between rs3217927 (located in the 3′-UTR region) and ALL, we forecasted the allele-specific targeting miRNAs interacted with complementary sequence motifs of *CCND2* rs3217927 [40]. Based on our bioinformatics analysis using miRanda and TargetScan Database (http://www.bioguo.org/miRNASNP/index.php), has-miR-922 and has-miR-4291 specifically binding the sequence surrounding the variant site came to our sights. Besides, we noticed that the Gibbs free energy was -19.30 kcal/mol for the rs3217927 A allele, and 0 kcal/mol for the G allele. These bioinformatics forecasts indicated that this SNP may change the conformation of the secondary structure of *CCND2* itself and may affect the binding affinity between *CCND2* mRNA and the miRNAs (has-miR-922 or has-miR-4291 or both), which may finally alter the expression of cyclinD2. But no obvious difference was found here indicates A to G mutation changed the expression level of CCND2 mRNA. The reason for this result may be that we only have 57 ALL patients with three genotypes of CCND2 to conduct PCR assay (37 genotype AA, 16 genotype AG and 4 genotype GG, respectively). There are not sufficient GG type samples to provide high certainty. A larger size of subjects is needed to repeat this part of experiment in the following research. Based on discussion above, it is proper to suspect that rs3217927 may influence miRNA biogenesis and function, and may contribute to susceptibility for childhood ALL. Of course, this is just one hypothesis of many unknown pathogenesis. Further functional evaluations in *vitro* and *vivo* with a larger size of samples are needed to confirm our conjecture.

Some limitations of this study should be addressed. Firstly, this study is hospital-based case-control designed, which may give rise to selection bias of subjects related with some particular genotypes. To minimize potential confounding bias, we adopt rigorous exclusion criteria about epidemiological design in recruitment of control subject which is frequency matched to cases by age, sex and ethnicity. The genotype frequencies of rs3217927 polymorphism among the controls in our study were consistent with the information provided by the HapMap Project. Secondly, when designing this experiment, we did notice that an independent validation cohort is necessary but our data was just over the minimum value of statistical power (709 cases and 709 controls), which was performed by Epicacl 2000 based on 1∶1 ratio of cases to controls, expected OR 1.35, proportion controls exposed 40.0%, thresholds set on 0.05 and 80% of the calculated degree of certainty. In order to have the most trustworthy relevance, we devoted all our samples into relevance verification. Although we hold the opinion that the current data in this manuscript is enough to analyze the risk of ALL, it will be ideal to carry on a two-stage model. And we are still gathering data. Besides, it is essential to validate our results in a larger size of subjects, which we believe should be at least 4044 subjects in total containing 2022 cases and 2022 controls. This assessment was performed using Epicacl 2000 based on 1∶1 ratio of cases to controls, expected OR 1.2, proportion controls exposed 35.0%, thresholds set on 0.05 and 80% of the calculated degree of certainty. Thirdly, the detailed epidemiologic data provided here is not adequate to evaluate gene-environment interaction. In order to provide more detailed interpretation of association between environmental toxic exposure and ALL, it would become an essential pre-work to acquire abundant epidemiologic exposure data and clinical information. Last but not least, our research about the gene susceptibility connected with childhood ALL is only limited on the statistics and epidemiology level and the further functional studies are warranted to validate our findings and reveal the underlying molecular mechanisms.

In conclusion, for the first time we found evidence that rs3217927 polymorphism in the cell cycle gene *CCND2* may be relevant to susceptibility of Childhood ALL in a Chinese population. Further validation in a larger sample size with diverse ethnic populations and functional evaluations in *vitro* and *vivo* are warranted.

## Supporting Information

Figure S1 The nucleotide localization of the CCND2 rs3217927 primer sequences.
(DOCX)

Table S1 Interaction analyses of CCND2 rs3217927 and parental drinking and house-painting.
(DOC)
