# Supplementary figures and images for: Association between the Polymorphism rs3217927 of CCND2 and the Risk of Childhood Acute Lymphoblastic Leukemia in a Chinese Population
Source: PLoS One. 2014 Apr 17;9(4):e95059. doi: 10.1371/journal.pone.0095059 (PMC3990598; doi:10.1371/journal.pone.0095059)

**Supporting information**

**Figure S1. The nucleotide localization of the CCND2 rs3217927 primer sequences**


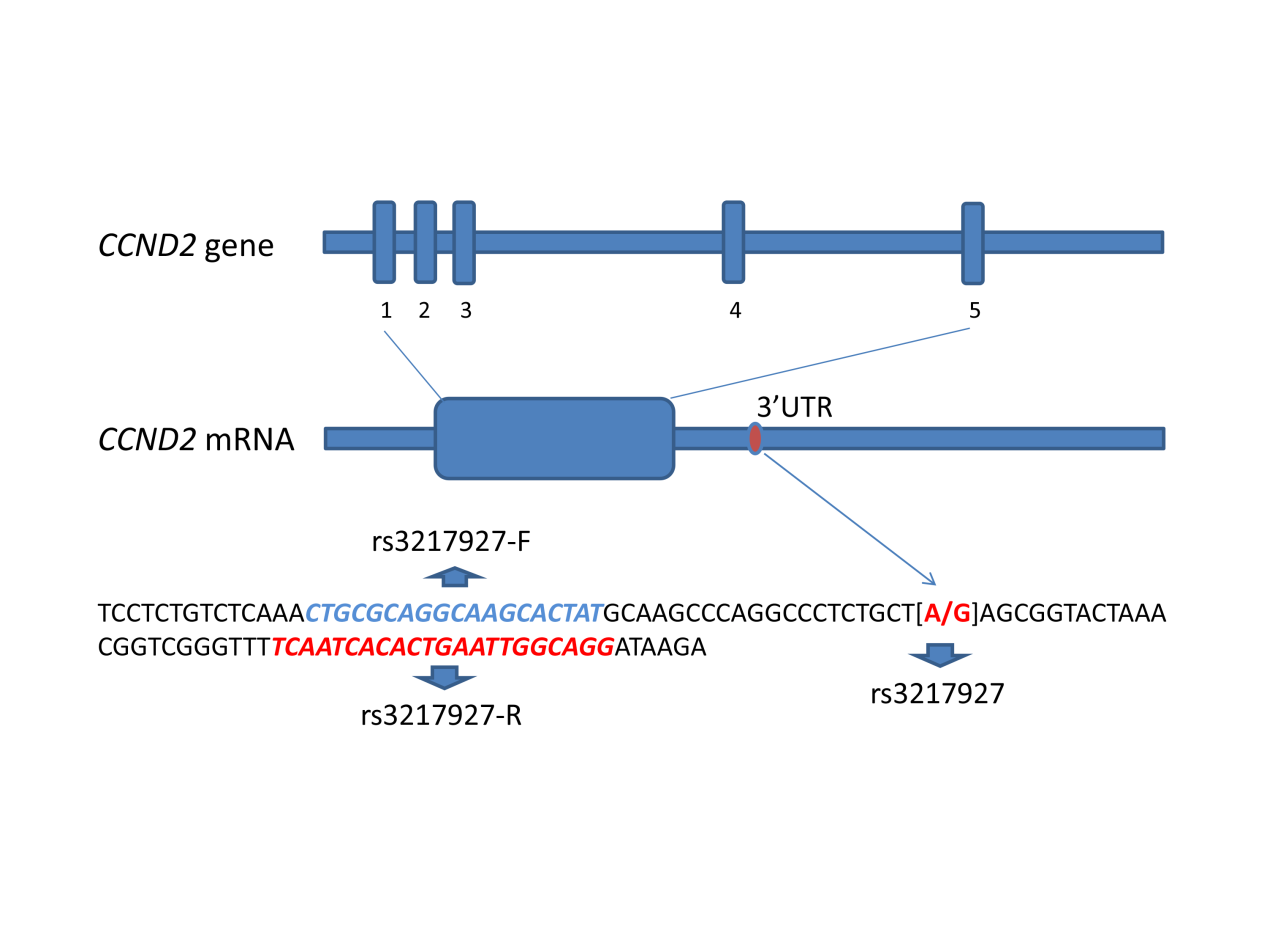

Supplement: Figure S1 — The nucleotide localization of the CCND2 rs3217927 primer sequences. (DOCX) [file pone.0095059.s001.docx]
