# Supplementary material for: Association between the Polymorphism rs3217927 of CCND2 and the Risk of Childhood Acute Lymphoblastic Leukemia in a Chinese Population
Source: PLoS One. 2014 Apr 17;9(4):e95059. doi: 10.1371/journal.pone.0095059 (PMC3990598; doi:10.1371/journal.pone.0095059)
Supplement: Table S1 — Interaction analyses of CCND2 rs3217927 and parental drinking and house-painting. (DOC) [file pone.0095059.s002.doc]

**Supporting information**

**Table S1. Interaction analyses of CCND2 rs3217927 and parental drinking and house-painting**

| **Status** | **Genotypes** | **Cases (n=753)** | |  | **Controls (n=1088)** | | ***Adjusted OR***  **(95% *CI*)^b^** | *P*^a^ |
| --- | --- | --- | --- | --- | --- | --- | --- | --- |
|  |  | n | % |  | n | % |  |  |
| **nondrinkers** | **AA/AG** | 471 | 62.5 |  | 838 | 78.0 | 1.00(reference) |  |
| **nondrinkers** | **GG** | 22 | 3.0 |  | 22 | 2.0 | 1.77 (0.96 3.23) | 0.066 |
| **drinkers** | **AA/AG** | 240 | 31.9 |  | 218 | 20.0 | 0.50 (0.22 1.12) | 0.095 |
| **drinkers** | **GG** | 20 | 2.6 |  | 10 | 1.0 | 0.73 (0.32 1.67) | 0.455 |
| **Interaction**  **(multiplicative)** |  |  |  |  |  |  |  | 0.465 |
|  |  |  |  |  |  |  |  |  |
| **nonpainting** | **AA/AG** | 457 | 60.7 |  | 779 | 71.6 | 1.00(reference) |  |
| **nonpainting** | **GG** | 26 | 3.5 |  | 24 | 2.2 | 1.73 (0.98 3.06) | 0.061 |
| **painting** | **AA/AG** | 254 | 33.7 |  | 277 | 25.5 | 0.47 (0.20 1.15) | 0.098 |
| **painting** | **GG** | 16 | 2.1 |  | 8 | 0.1 | 0.75 (0.30 1.86) | 0.529 |
| **Interaction**  **(multiplicative)** |  |  |  |  |  |  |  | 0.752 |

^a^Two-sided chi-square test for either genotype distribution or allele frequencies between cases and controls.

^b^Adjusted for age, gender, parental drinking status, parental smoking status, and house painting status.
